# Sedentary time by occupation in a nationally representative Japanese population: a descriptive study using the National Health and Nutrition Survey

**DOI:** 10.1093/joccuh/uiag003

**Published:** 2026-01-23

**Authors:** Noritoshi Fukushima, Hiroyuki Kikuchi, Shiho Amagasa, Masaki Machida, Takashi Nakagata, Rei Ono, Shigeru Inoue

**Affiliations:** Department of Preventive Medicine and Public Health, Tokyo Medical University, Tokyo, Japan; Department of Preventive Medicine and Public Health, Tokyo Medical University, Tokyo, Japan; Department of Preventive Medicine and Public Health, Tokyo Medical University, Tokyo, Japan; Graduate School of Public Health, Teikyo University, Tokyo, Japan; Department of Preventive Medicine and Public Health, Tokyo Medical University, Tokyo, Japan; Center for Physical Activity Research, National Institutes of Biomedical Innovation, Health and Nutrition, Osaka, Japan; Department of Social Science, National Center for Geriatrics and Gerontology, Aichi, Japan; Department of Preventive Medicine and Public Health, Tokyo Medical University, Tokyo, Japan

**Keywords:** guidelines, office workers, sedentary behavior, surveillance, workplace health promotion

## Abstract

**Objectives:**

Prolonged sitting time is gradually being recognized as detrimental to health. As technological advances have made workplaces increasingly sedentary, describing sitting time by occupation is useful for identifying at-risk groups and promoting occupational health. This study aimed to elucidate the differences in sedentary time according to occupation using a nationally representative sample.

**Methods:**

Self-reported total sedentary (sitting or lying down during waking hours) time was obtained from the 2013 National Health and Nutrition Survey. Occupations were classified as professionals, managers, clerks, sales, service, protective services, agricultural/forestry/fishery workers, transport/machine operators, manufacturing/construction/cleaning laborers, homemakers, and unemployed individuals. Sedentary time by occupation was compared using an analysis of covariance adjusted for age and sex.

**Results:**

A total of 4071 workers aged 20-64 years were analyzed. Age-adjusted sedentary time was longer in men than in women. The age-adjusted sedentary time among different occupations ranged from 280 to 499 min/d. The longest age-adjusted sedentary time was observed among clerks (499 min/d), followed by managers (437 min/d). In contrast, the shortest sedentary time was observed among agricultural/forestry/fishery workers (280 min/d). Clerks and managers had longer sedentary time on weekdays than on days off, whereas other occupations, including homemakers, did not.

**Conclusions:**

Sedentary time differed substantially among occupations, with a variation of approximately 3.5 h/d. Prolonged sedentary time, a known health risk, may represent a substantial occupational exposure, particularly among clerical workers. Occupational health staff should monitor workers’ sedentary time to promote better occupational health outcomes.

## Teaser

**Key points**

***What is already known on this topic***:

Physical activity levels among the Japanese working population are decreasing. Prolonged sedentary time is associated with adverse health outcomes; therefore, sedentary occupations are an important public health concern.

***What this study adds***:

This study presents self-reported sedentary time by occupation using a nationally representative sample from the National Health and Nutrition Survey of Japan. The most sedentary occupations were clerks, followed by managers, whereas the least sedentary occupations were agriculture/forestry/fishery.

***How this study might affect research, practice, or policy***:

Clerks were more exposed to the risk of adverse health outcomes caused by prolonged sedentary time than any other occupation. Workplace interventions to reduce occupational sedentary time are warranted, especially among clerical workers.

## 1. Introduction

Sedentary behavior (SB) is associated with adverse health outcomes;^1^ however, it remains highly prevalent in daily life, including leisure time and occupational domains.^2,3^ As the physical activity (PA) component of work has decreased, many occupations are becoming increasingly sedentary owing to technological advancements.^4–6^ The total amount of daily step-defined PA has been declining in most occupations in the past 2 decades.^7^ Therefore, prolonged sitting among workers is an important public health concern.^4^

Recent guidelines on PA emphasize the importance of reducing excessive SB in addition to accumulating moderate-to-vigorous-PA.^8–10^ The 2020 World Health Organization guidelines recommend that adults limit the amount of sedentary time.^8^ Additionally, the current Japanese guidelines, the Physical Activity Guide for Health Promotion 2023,^10^ have introduced recommendations to avoid SB, such as prolonged sitting. Detailed descriptions of sitting time across all age groups and working populations, based on national samples, are informative and useful under current Japanese PA guidelines. However, data on sitting time in the Japanese population are limited, especially among working adults by occupation.^11^ Understanding how much time working adults spend in SB is crucial for promoting occupational health and identifying target groups for intervention.^12^

In Japan, the 2013 National Health and Nutrition Survey (NHNS-J) provides data on sitting time and occupational classifications using a nationally representative sample. Moreover, data on sedentary time during weekdays and days off are available from the NHNS-J. Time is used differently between workdays and free days; furthermore, previous studies reported different SB patterns during workdays and free days between white- and blue-collar workers,^13^ with different levels of sedentary time among occupations depending on the day.^14^ However, the difference in sedentary time between weekdays and days off within each occupational category has not been quantitatively evaluated.

Therefore, this study aimed to evaluate sedentary time stratified according to sex, age, and occupational classification, and compare sedentary time between weekdays and days off per occupation.

## 2. Methods

### 2.1. Participants and data collection

The NHNS-J is a cross-sectional household interview and examination survey comprising 3 parts: (1) physical examination, (2) nutritional assessment, and (3) lifestyle assessment. Although the most recent NHNS-J survey collected data on sitting time in 2017, the responses were categorical (<3 hours, 3-8 hours, or ≥8 hours).^15^ In contrast, the 2013 survey measured sedentary time as a continuous variable,^15^ providing more detailed and informative data; therefore, we used data from 2013 when a questionnaire on sedentary time was administered in the lifestyle assessment section. The detailed participant sampling design for the NHNS-J has been described elsewhere.^15,16^ Briefly, the NHNS-J begins by randomly selecting 300 census units from census enumeration areas previously selected as part of the Comprehensive Survey of Living Conditions of the People on Health and Welfare.^17^ Each census unit included approximately 20 households; 5204 households were sampled, and 3493 households responded in this survey year.^16^ Individuals aged ≥20 years were invited to participate in the lifestyle assessment part of the NHNS-J. In the descriptions of sedentary time by age group and sex, we included all samples ranging from 20 to 101 years of age. In contrast, in the description of sedentary time by occupation as the main purpose of this study, we included working adults aged 20-64 years. We excluded pregnant or postpartum women, students, those who reported 0 or >960 min/d (>16 h/d) of sedentary time, or those with missing data. The threshold of 16 h/d was based on the assumption, described in the Guidelines for Data Processing and Analysis of the IPAQ,^18^ that individuals spend approximately 8 hours sleeping each day. Under this assumption, the remaining 16 hours represent the maximum waking time; therefore, reported sedentary time exceeding this limit was excluded.

### 2.2. Sedentary time

Sedentary time was assessed using the NHNS-J questionnaire as follows: “On average, how much time do you spend sitting or lying down during the day?” Sitting or lying down time included the time spent sitting at a desk or computer (including work, study, and reading), watching TV, sitting and conversing, driving a car (or riding in a car), and sitting on a train, whereas sleep duration was not included. The questionnaire on sitting time was originally developed for the NHNS-J with some modifications of the International Physical Activity Questionnaire Short Form.^15,19^ The NHNS-J questionnaire assessed sedentary time during weekdays and days off separately. The sedentary time (hours and minutes per day) was reported on weekdays and days off. Weighted sedentary time (min/d) was calculated as follows: (weekday sitting min × 5 weekdays + days off sitting min × 2 days off)/7.

### 2.3. Occupational classifications

The occupational classifications in the NHNS-J questionnaire included professionals, managers, clerks, sales, service, protective services (eg, police officers, security guards), agricultural/forestry/fishery workers, transport/machine operators, manufacturing/construction/cleaning laborers, homemakers, and unemployed individuals.^7,20^ The unemployed category included participants unable work because of illness or disability, although information on the specific condition or severity was not available. Missing data on occupation were categorized into “missing (unknown).”

### 2.4. Statistical analysis

Continuous data are presented as means with SDs, and categorical data as frequencies and proportions. Comparisons of sedentary time among occupations were made using analysis of covariance adjusted for sex and age. We examined the potential nonlinear association between age and sedentary time by comparing model fit with and without a quadratic term of age, using the *R*^2^ change and the *F*-change test. Sedentary times on weekdays and days off by occupation were compared using a paired *t*-test. All statistical analyses were performed using IBM SPSS Statistics version 28 (IBM Corp, Armonk, NY, USA), and significance was set at *P* < .05.

### 2.5. Ethics approval and consent to participate

This study was a secondary analysis of anonymized data obtained by the Ministry of Health, Labor and Welfare (MHLW). The survey was conducted according to Japan’s Health Promotion Law. The Ministry of Internal Affairs and Communications of Japan reviewed and approved the survey protocols, and informed consent was obtained from all participants. The use of individual raw data from the NHNS-J in 2013 was approved by the MHLW through official application procedures under Article 33 of the Japanese Statistics Act. The Ethics Committee of Tokyo Medical University confirmed that this study was based on the secondary use of public data and that there was no need for ethical approval.

## 3. Results

**Figure 1:**
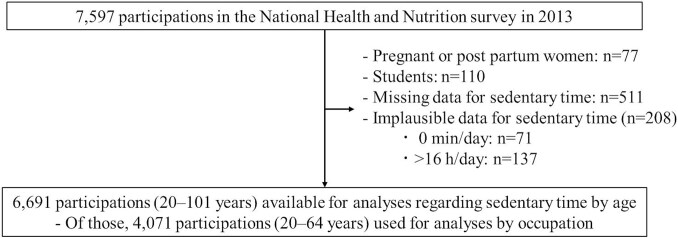
Participant flow.

Figure 1 illustrates the participant flow. After excluding 906 participants (pregnant or postpartum women, *n* = 77; students, *n* = 110; missing data on sedentary time, *n* = 511; and inappropriate sitting time, *n* = 208), 6691 participants aged 20-101 years were included and analyzed by age categories. Of the 6691 participants, 4071 workers aged 20-64 years were analyzed by occupational categories. The participant characteristics are shown in Table 1. Among the 6691 participants, the mean (SD) age was 57.1 (17.2) years, and 46.5% were men. Participants aged 60-69 years were the most represented (22.0%), whereas those aged 20-29 years were the least represented (6.9%) (Table 1). Regarding occupational categories (*n* = 4071), 3020 (74.2%), 613 (15.1%), and 229 (5.6%) were working with income, homemakers, and unemployed, respectively (Table 1). Among participants with income, clerks were the most common (15.4%), whereas protective workers were least common (1.0%) (Table 1).

**Table 1:** Characteristics of NHNS-J (*n* = 6691).

Characteristic	
**Sex**	
**Men**	3110 (46.5)
**Women**	3581 (53.5)
**Age, mean [SD], y**	57.1 [17.2]
**Age categories, y**	
**20-29**	465 (6.9)
**30-39**	804 (12.0)
**40-49**	1064 (15.9)
**50-59**	1025 (15.3)
**60-69**	1473 (22.0)
**70-79**	1263 (18.9)
**≥80**	597 (8.9)
**Occupation classification** ^**a**^	
**Working with income**	
**Professionals**	606 (14.9)
**Managers**	246 (6.0)
**Clerks**	627 (15.4)
**Sales workers**	312 (7.7)
**Service workers**	492 (12.1)
**Protective services workers**	39 (1.0)
**Agricultural, forestry, and fishery workers**	65 (1.6)
**Transport/machine operators**	76 (1.9)
**Manufacturing/construction/cleaning laborers**	557 (13.7)
**Homemakers**	613 (15.1)
**Unemployed individuals**	229 (5.6)
**Unknown (missing)**	209 (5.1)

Abbreviation: NHNS-J, National Health and Nutrition Survey–Japan.

Data are expressed as *n* (%) unless otherwise indicated.

^a^Participants aged 20-65 years are analyzed to examine the sedentary time by occupation.

Descriptions of weighted sedentary time by age group are presented in Table 2. The sedentary time in all ages was estimated as 395 min/d (95% CI, 390-400), and men spent more time sitting than did women (405 min/d for men and 385 min/d for women) (Table 2). The median sedentary time in the total sample was 360 min/d (interquartile range: 244-514 min/d) (Table S1). In men, sedentary time gradually increased toward older age (373 min/d in those aged 20-29 years to 441 min/d in those aged ≥80 years). In women, sedentary times in the 20-29 years and ≥80 years categories were significantly longer than those in any other age category. In both sexes, a distinct increase in sedentary time was observed in the age category of ≥80 years (Table 2).

**Table 2:** Weighted sedentary time by sex and age categories.

	Total sample		Men		Women	
	Estimated mean	95% CI	Estimated mean	95% CI	Estimated mean	95% CI
**All ages**	395	390-400	405	398-412	385	379-392
**Age categories, y**						
** 20-29**	397^g^	379-415	373^d^^,^^f^^,^^g^	347-399	420^a^^,^^b^^,^^c^^,^^d^^,^^e^^,^^f^^,^^g^	395-445
** 30-39**	381^g^	367-394	396^g^	376-416	366^a^^,^^g^	347-385
** 40-49**	388^g^	376-400	399^g^	382-417	377^a^^,^^g^	361-393
** 50-59**	395^g^	383-407	415^a^	397-433	376^a^^,^^g^	359-392
** 60-69**	384^g^	374-394	401^g^	386-416	368^a^^,^^g^	355-382
** 70-79**	395^g^	384-406	410^a^^,^^g^	393-426	382^a^^,^^g^	367-396
** ≥80**	456^a^^,^^b^^,^^c^^,^^d^^,^^e^^,^^f^	440-472	441^a^^,^^b^^,^^c^^,^^d^^,^^e^^,^^f^^,^^g^	416-467	463^a^^,^^b^^,^^c^^,^^d^^,^^e^^,^^f^^,^^g^	443-484

Weighted sedentary time (min/d) is calculated as follows: (weekday sitting min × 5 weekdays + weekend day sitting min × 2 weekend days)/7.

^a^ *P* < .05 vs 20-29 years.

^b^ *P* < .05 vs 30-39 years.

^c^ *P* < .05 vs 40-49 years.

^d^ *P* < .05 vs 50-59 years.

^e^ *P* < .05 vs 60-69 years.

^f^ *P* < .05 vs 70-79 years.

^g^ *P* < .05 vs ≥80 years.

When comparing sedentary time among occupations, including the quadratic age term significantly improved model fit compared with using only a linear term (Δ*R*^2^=0.003, *P* < .001). Therefore, the quadratic term of age was retained in the final analysis. As a result, agricultural/forestry/fishery workers spent the least sedentary time, whereas clerks spent the most (Table 3). Compared with agricultural/forestry/fishery workers, clerks spent more than 3.5 h/d sitting. None of the participants among women aged <65 years were engaged in protective work.

**Table 3:** Comparisons of weighted sedentary time by occupation.

	Total sample		Men		Women	
	Estimated mean	95% CI	Estimated mean	95% CI	Estimated mean	95% CI
**Professionals**	392^c,e,g,i,k^	377-407	432^c^^,^^d^^,^^e^^,^^g^^,^^i^	411-452	346^b,c,g,k^	324-367
**Managers**	437^c,d,e,g,i,j^	415-460	434^c^^,^^d^^,^^e^^,^^g^^,^^i^	410-458	465^a^^,^^e^^,^^g^	402-529
**Clerks**	499^a^^,^^b^^,^^d^^,^^e^^,^^f^^,^^g^^,^^i^^,^^j^^,^^l^	483-514	499^a^^,^^b^^,^^d^^,^^e^^,^^f^^,^^g^^,^^i^^,^^j^^,^^l^	474-524	495^a^^,^^d^^,^^e^^,^^g^^,^^i^^,^^j^^,^^k^^,^^l^	476-513
**Sales workers**	355^b,c,g,h,k^	336-374	355^a,b,c,k^	328-383	353^c^^,^^g^^,^^k^	326-380
**Service workers**	323^a,b,c,h,j,k,l^	307-338	318^a^^,^^b^^,^^c^^,^^h^^,^^k^^,^^l^	291-345	322^b,c,j,k,l^	303-341
**Protective services workers^m^**	373^c^	316-429	375^c^	318-433	NA^m^	NA^m^
**Agricultural, forestry, and fishery workers**	280^a,b,c,d,h,j,k,l^	253-307	307^a^^,^^b^^,^^c^^,^^h^^,^^k^^,^^l^	273-341	244^a,b,c,d,i,j,k,l^	198-289
**Transport/machine operators**	433^d,e,g,i^	394-473	437^e^^,^^g^^,^^i^	396-478	374	160-587
**Manufacturing/construction/cleaning laborers**	339^a,b,c,g,h,j,k,l^	324-354	333^a^^,^^b^^,^^c^^,^^h^^,^^k^^,^^l^	315-351	364^c,g,k^	334-393
**Homemakers**	383^b,c,e,g,i,k^	372-394	358^c^^,^^k^	313-403	379^c,e,g,k^	368-389
**Unemployed individuals**	436^a,c,d,e,g,i,j^	424-448	446^d,e,g,i,j^	430-461	433^a,c,d,e,g,i,j^	413-453
**Missing (unknown)**	403^c,e,g,i^	382-424	424^c,e,g,i^	393-455	384^c^^,^^e^^,^^g^	356-411

Abbreviation: NA, not applicable.

Weighted sedentary time is adjusted for sex and age using analysis of covariance.

^a^ *P* < .05 vs Professionals.

^b^ *P* < .05 vs Managers.

^c^ *P* < .05 vs Clerks.

^d^ *P* < .05 vs Sales workers.

^e^ *P* < .05 vs Service workers.

^f^ *P* < .05 vs Protective service workers.

^g^ *P* < .05 vs Agricultural, forestry, and fishery workers.

^h^ *P* < .05 vs Transport/machine operators.

^i^ *P* < .05 vs Manufacturing/construction/cleaning laborers.

^j^ *P* < .05 vs Homemakers.

^k^ *P* < .05 vs Unemployed individuals.

^l^ *P* < .05 vs Missing (unknown).

^m^None of the participants among women aged <65 years were engaged in protective work.

**Figure 2:**
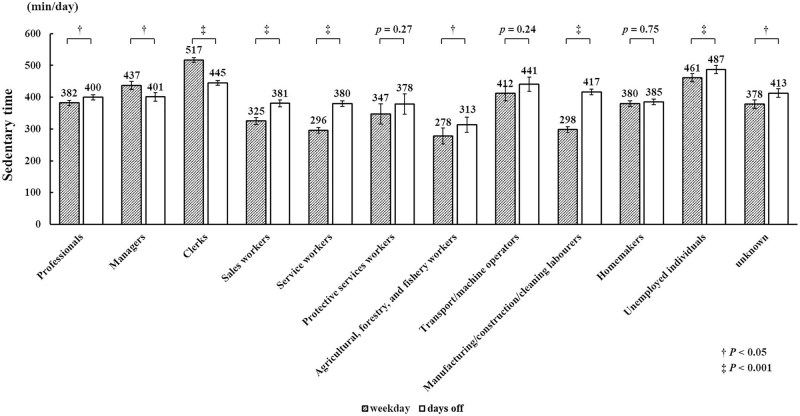
Comparisons of sedentary time by occupations between weekdays and days off.

Clerks had the longest sedentary time on weekdays and the second longest sedentary time on their days off. No significant differences in sedentary time between weekdays and days off were observed among protective workers, transport/machine operators, and homemakers. In contrast, the remaining occupations showed differences in sedentary time between weekdays and days off (Figure 2). Among these, only managers and clerks spent more time sitting on weekdays than on days off, whereas other occupations spent more time sitting on days off than on weekdays.

## 4. Discussion

Using a nationally representative sample, we observed considerable differences in sedentary time among occupations, ranging from 280 to 499 min/d. Clerks and managers were exposed to longer sedentary time than those in other occupations with income. Clerks and managers spent more time sitting on weekdays than they did on days off. Specifically, clerk is a common occupation among Japanese workers,^21^ indicating a need for interventions that help to reduce sedentary time during workdays.^13^

Among studies comparing sitting time per occupation, Jans et al^11^ previously reported the differences in sitting time among occupations by business sectors in the Dutch working population (*n* = 7720 in a representative sample of Dutch households). Our findings are in line with those of that previous study,^11^ in which clerks and managers spent more time sitting than did workers of other occupations, with those in agricultural occupations exhibiting the least sitting time. Although differences in questionnaires between studies warrant a cautious interpretation of sitting time, these studies using national samples showed the robustness of data reporting clerks sitting for a relatively long time compared with other occupations. The mean sitting time of clerks in the Japanese population was slightly longer but close to that in the Dutch population (496 vs 477 min/d), suggesting that nearly half of the clerks would sit for more than 8 hours in both countries. In this regard, the Canadian PA guidelines explicitly state that sedentary time should be limited to 8 hours or less^9^; hence, reducing sitting time may be particularly important for clerical workers.

Furthermore, the study by Jans et al^11^ did not report differences in sitting time between weekdays and days off. The present study adds to the literature by comparing sedentary time between weekdays and days off among different occupations. We observed that clerks not only had the second longest sedentary time on days off but also spent about 1 more hour sitting on weekdays than on days off compared with other participants. In addition, the differences in weighted sedentary time among occupations reached approximately 3.5 hours. Each 1-hour increment in daily sitting time is associated with a 4% increase in the risk of cardiovascular disease and a 3% increase in all-cause mortality.^22^ In addition, So et al^14^ reported that replacing 1 h/d of sitting with an equal amount of standing/walking at the workplace was associated with a 4% decrease in the risk for dyslipidemia and a 7% decrease in the risk for heart disease among Japanese workers. Moreover, a previous study reported that most of the differences in SB time among occupations occurred during working hours on weekdays;^23^ therefore, reducing sitting time during working hours would be an important interventional strategy, especially for clerks. Environmental support, motivational strategies, and multiple-component interventions, including using digital elements, are effective in reducing workplace sitting time, especially for office workers.^4,24,25^

In addition, we found that managers had the second-longest sedentary time among income-related occupations. This is partly because managers tend to spend more time at the workplace owing to longer overtime hours compared with nonmanagers.^26^ This may be related to the fact that managers are more likely to have unhealthy lifestyle habits (e.g. lacking regular exercise) and unfavorable cardiovascular risk factors.^26^ Moreover, prolonged sitting time is associated with an increased risk of depression and suicidal ideation among workers, especially managers,^27–29^ suggesting that managers who spend a long time sitting should pay attention to mental health.

In this study, we described the sitting time among adults in all age groups. The current Japanese PA guidelines encourage the population to “break up prolonged sitting and add 10 more minutes of PA to their daily lives.”^10^ Reference data describing sitting time in the Japanese population are therefore informative. However, a previous large-scale international comparative study across 20 countries, including Japan, reported self-reported national sitting times in individuals aged 20-65 years.^30^ Given that Japan has one of the most rapidly aging populations in the world,^31^ it is useful to demonstrate sitting time not only in the working population but also in older adults. Indeed, we were able to provide further evidence on sedentary time in older adults using national samples. Moreover, sedentary time in this study was shorter than that reported for Japan in a previous international comparative study (median in total sample: 360 vs 420 min/d).^30^ This discrepancy may be partly explained by selection bias, because participants in the aforementioned study were recruited from 22 universities and 6 worksites.^32^ Office workers tend to spend more time sitting;^23,33^ hence, sedentary time may have been overestimated in that study.

Here, we observed that sedentary time among women showed a U-shaped pattern across age categories, whereas that among men gradually increased with age. This nonlinear pattern among women is consistent with the findings of Harrington et al^34^ using NHANES data, in which sitting time increased with age but was lower among middle-aged women than among older women. In our study, sedentary time among women aged 20-29 years was longer than that in middle-aged groups, and it tended to increase again toward older age. Working status (eg, full-time employment) and marital status (eg, unmarried status) may be associated with long sitting time,^35–37^ especially among women. Although data on marital status are not available, given that more Japanese women participate in the workforce and young Japanese women tend to marry later in life,^38,39^ these sociodemographic factors might be associated with longer sitting times among young women. A previous review reported that associations between age, sex, and sedentary behavior were not consistent across Asian populations;^37^ therefore, further research is warranted to describe age- and sex-specific patterns more accurately. Our study contributes to this by providing nationally representative evidence from Japan.

A strength of our study is that we presented sedentary time by occupation—including homemakers—using a nationally representative sample. Both occupational and household PA levels have declined over the past half-century;^5,40^ hence, prolonged sitting has become an increasingly important health concern. Our findings provide useful information to support health promotion efforts targeting working population.

However, this study has some limitations. First, because sedentary time was measured using a self-reported questionnaire, recall bias could have introduced a tendency to underestimate sitting time.^41^ Second, the response rate was 67.1%,^16^ and there were no data from nonrespondents, which hampered the discussion on selection bias. Third, only the total sedentary time was reported; therefore, we could not further categorize this time into the occupational, leisure, or transport domains.^42^ Fourth, the classifications of occupational and industry categories differed across studies,^14,37^ making direct comparisons difficult. Despite these limitations, data on sedentary time according to occupation in a nationally representative Japanese population are valuable.

## 5. Conclusions

Sedentary time differed widely among occupations, ranging from 280 to 499 min/d. Furthermore, sitting is identified as an inherent occupational feature of clerical work in office settings. Therefore, prolonged SB may be one of the harmful occupational hazards related to future health outcomes, particularly among clerical workers. Workplace interventions to reduce sitting time in clerical occupations are warranted.

## Supplementary Material

uiag003_Supplementay_materials
