# Supplementary material for: Sedentary time by occupation in a nationally representative Japanese population: a descriptive study using the National Health and Nutrition Survey
Source: J Occup Health. 2026 Jan 23;68(1):uiag003. doi: 10.1093/joccuh/uiag003 (PMC13082221; doi:10.1093/joccuh/uiag003)
Supplement: uiag003_Supplementay_materials [file uiag003_supplementay_materials.pdf]

Supplementary Table 1 The 25th percentile, 50th percentile, and 75th percentile of weighted sedentary time (min/day) by gender.

	Total sample			Men			Women		
	25%tile	50%tile	75%tile	25%tile	50%tile	75%tile	25%tile	50%tile	75%tile
All ages	244	360	514	257	360	540	240	351	489
Age categories, years									
20–29	266	360	506	252	351	479	271	377	559
30–39	231	342	506	234	360	516	231	326	480
40–49	242	351	514	249	360	531	240	343	480
50–59 years	257	360	514	257	386	553	249	341	480
60–69	240	360	480	257	366	518	240	351	480
70–79	240	360	501	240	360	540	240	354	480
≥80	300	420	600	274	420	600	300	463	600
Occupation classifications*									
Working with income									
Professionals	231	351	514	266	394	591	212	317	429
Managers	291	407	582	281	403	583	304	454	566
Clerks	339	489	626	326	507	634	351	471	621
Sales workers	214	319	427	214	334	463	214	309	411
Service workers	206	300	394	208	300	415	206	300	394
Protective services workers	157	386	514	157	386	514	N.A*	N.A*	N.A*
Agricultural, forestry, and fishery workers	197	300	360	193	309	386	193	272	300
Transport/machine operators	291	420	564	290	420	574	334	385	-
Manufacturing/construction/cleaning laborers	231	317	422	231	309	420	237	345	455
Homemakers	251	334	480	281	360	482	249	334	480
Unemployed individuals	300	446	600	300	448	600	249	446	729
Unknown (missing)	240	360	514	251	386	551	214	351	471

*None of the participants among women aged <65 years are engaged in protective work.
